# A Visit to the Hospital at Zurich University

**Published:** 1890-04-12

**Authors:** 


### A Visit to the Hospital at Zurich University.

The universities of Central Europe are all in a sense members of one union. This embraces the two-and-twenty universities of the German Empire, six in Switzerland, seven in Austria-Hungary, and one in Russia. In them all, with one or two exceptions—as, for instance, Geneva, where lectures are in French, and Lemburg, where they are in Polish—German is the language spoken and German methods prevail. Students of all and each of them have the privilege of passing from one to another and taking up the thread of their curriculum where they had left it off.

From Freiburg one could have passed north to visit the famous Heidelberg, or journeyed some way east to Munich, where in a town of 250,000 inhabitants there were last summer 1,500 students of medicine. Leaving these, and other yet more famous seats of learning belonging to this system, such as Berlin and Vienna, which, with Paris, are the medical schools of the Continent par excellence, for the present one proceeded viâ Basel to Switzerland. Basel, with the Rhine already a wide river cutting it in two, with French over one hotel and German over the next, has a university of all the faculties, 90 teachers and over 400 students. But across the frontier, on the lovely lake of the same name, familiar to many English tourists, is a more celebrated university town—Zurich. There are 567 students studying in its four faculties. Of these, 39 study Evangelical theology, 56 law and jurisprudence, 131 philosophy, science, and mathematics, and 288 medicine, surgery, and pharmacology. Reserving for the future a more careful and detailed account of the appliances and methods of the scientific instruction imparted to medical students, it is proposed in this place to devote a few paragraphs to the hospital, which is the seat of their clinical instruction.

On coming to the hospital one could not help enquiring whether a new and fine building in process of erection not far off was not the new hospital—it proved to be the new Hygienic and Physiological Institute. And the first almost unconscious impression that the provision for the care of patients was inferior to that for imparting knowledge was strengthened on finding that the arrangements in the wards were curiously behind the spirit of the age in certain important particulars. The nurses in woollen gowns and large aprons, with stout youths to act as assistants, somewhat recalled the days of Mrs. Gamp. The corridors were paved with bare flags, the walls in part whitewashed, and many of the wards a little mean in appearance. The whole 150 medical patients were under the charge of one physician, who acted likewise in the schools as professor of clinical medicine and of the special pathology and theory of nervous and infectious diseases. Moreover the 150 surgical beds were under the care of a gentleman who was likewise professor of surgery, and who gave demonstrations during the session upon Hernia. The 80 beds for diseases of women are in a separate building.

The temperatures stood noted in chalk on blackboards over the patients' beds, under the patients' names written large. The nationality of the patients is extraordinarily various; as various as the nationality of the coins which one may receive on paying a bill. As one finds in his hand French, Swiss, Italian, and even Greek money; so one finds in these beds for the sick Russians, Italians, Germans, even Dutch—but there was no Englishman, nor even a Scotchman, who is currently supposed to be everywhere. A curious hieroglyphic is drawn on the medicine bottles containing poison, and does the work of the poison labels common among ourselves. It consists of a skull with cross bones, such as we are accustomed to associate with a certain variety of threatening letter in England.

The better wards are fairly roomy and lofty, containing twelve beds each. The bedsteads are entirely metal as high as the mattresses, which rest upon a chainwork frame, stretching between the posters. The hospital shares with private dwellings in this part of the Continent a substitute for blankets unknown to us at home. Upon the sheet and quilt which cover the patient rests a large bag of feathers or stuff, square in form, flattened out so as to clothe the body lying beneath. This arrangement is sufficiently warm so long as it keeps in position, but is very liable to roll off upon the floor. The stand by the bedside containing the patient's effects in a cupboard carries also his medicine and his wine. The latter was ready at every bedside in a litre bottle—a litre is about a pint and three-quarters. It is the vin ordinaire, red wine, of these parts, containing, it will be found, very little intoxicating spirit. Heating is partly effected by stoves placed towards the centre of the department, whose winding chimneys utilise all the warmth which the wooden fuel furnishes. The floors are of wooden boards, worked into patterns, which are then polished with turpentine mixture. Among scientific apparatus are comparative colours for reference in describing the colour of the urine. There are nine shades for comparison, varying from yellow to brown-black. Esbach's solutions for exact determination of albumen are in use. Two varieties of apparatus for treating chest cases with rarified and compressed—not, however, medicated—air have fallen out of favour. As regards diet, breakfast is taken at half-past six a.m., and consists of coffee with bread; at half-past nine the patients partake of bread and bouillon; at twelve noon they have soup with meat and vegetables; at half-past three p.m. coffee; and at half-past six p.m. soups of various kinds. The diet in fever cases is confined to bouillon, which may contain three or four eggs, milk, and as much as half a litre of wine.

Having the opportunity of going round the wards with the professor of medicine, one had occasion to observe that the pleximeter was employed in percussion. The only noteworthy clinical cases were the frequent patients suffering from influenza, one or two remarkable cases of paralysis agitans, treated by suspension, several of diabetes insipidus, one doing well with antipyrine 45 grains daily, and a case of nephritis in the young subject, progressing with a prescription containing phosphoric acid. There was a case of disease of the left peroneal nerve with extensive nutritive disturbance in the region supplied by it, and a rare case of sclerodermia. A case of cardiac valvular insufficiency was being treated with acid phosphoric dil., calomel, and digitatis.

On the highest, that is the second flat, were some very comfortable wards for paying patients. An influenza patient, surrounded by every comfort, was being nursed by his wife. The same arrangement obtains at Freiburg.

In another building all cases of typhoid and tuberculosis are isolated. Typhoid fever is here termed typhus abdommalis: typhus proper is very rare. Among the cases of tuberculosis was one of peculiar interest, in the respect that the patient was ruminant—that is, brought up his food to be chewed a second time, after having once swallowed it. The fever patients are treated by cold baths, and phenacetin in doses of 15 gr. as an antipyretic. The baths are upon the same plan as those in use at Freiburg, but are not administered so frequently—at most twice a day.

The house physician starts upon his round at eight a.m., and the professor follows him at nine a.m. The latter is exemplary in dealing with malingerers. The frankness of his rebuke of a patient of this class is oftenest toned down among ourselves. "You were here for the whole of last year," said he, after sounding him. "You wish to stay here again. You find the hospital agreeable, and you desire to make the most of it. I am now going to discharge you, and the sooner you return home the better." He turned away, signed the needful papers, and left the patient to the opinion of the bystanders; but possibly to the sympathy of some, at least, of his companions in the ward.

